# High-Yield Production of Selected 2D Materials by Understanding Their Sonication-Assisted Liquid-Phase Exfoliation

**DOI:** 10.3390/nano11123253

**Published:** 2021-11-30

**Authors:** Freskida Goni, Angela Chemelli, Frank Uhlig

**Affiliations:** Institute of Inorganic Chemistry, Faculty of Technical Chemistry, Chemical and Process Engineering, Biotechnology, Graz University of Technology, Stremayrgasse 9, 8010 Graz, Austria; freskida.goni@tugraz.at (F.G.); frank.uhlig@tugraz.at (F.U.)

**Keywords:** 2D materials, liquid-phase exfoliation, high-yield production, graphene, boron nitride nanosheets, molybdenum disulfide nanosheets

## Abstract

Liquid-phase exfoliation (LPE) is a widely used and promising method for the production of 2D nanomaterials because it can be scaled up relatively easily. Nevertheless, the yields achieved by this process are still low, ranging between 2% and 5%, which makes the large-scale production of these materials difficult. In this report, we investigate the cause of these low yields by examining the sonication-assisted LPE of graphene, boron nitride nanosheets (BNNSs), and molybdenum disulfide nanosheets (MoS_2_ NS). Our results show that the low yields are caused by an equilibrium that is formed between the exfoliated nanosheets and the flocculated ones during the sonication process. This study provides an understanding of this behaviour, which prevents further exfoliation of nanosheets. By avoiding this equilibrium, we were able to increase the total yields of graphene, BNNSs, and MoS_2_ NS up to 14%, 44%, and 29%, respectively. Here, we demonstrate a modified LPE process that leads to the high-yield production of 2D nanomaterials.

## 1. Introduction

Two-dimensional (2D) nanomaterials have gained worldwide attention in recent years because of their outstanding properties due to their structure and dimensionality. Graphene was the first 2D material that was successfully isolated and studied in 2004 by Andre Geim and Konstantin Novoselov [1]. Graphene consists of sp^2^-hybridised carbon atoms that are hexagonally arranged in a honeycomb lattice. This unique structure is responsible for many of graphene’s excellent mechanical, electrical, thermal, and optical properties [1,2,3,4,5,6,7,8,9,10]. Due to these properties, graphene can be used in a variety of applications, ranging from nanoelectronics and energy storage to sensors and medicine [8,9,11,12,13,14,15,16]. Over the last decade, there has been an increasing interest in other 2D materials as well, e.g., hexagonal boron nitride (h-BN) [17,18], transition metal dichalcogenides (TMDs such as MoS_2_, TiS_2_, TaS_2_, WS_2_, etc.) [19,20], layered metal oxides [21,22,23], etc. Boron nitride nanosheets (BNNSs) have a similar structure to that of graphene, but this material consists of alternating boron and nitrogen atoms instead of carbon atoms. Similar to graphene, BNNSs exhibit outstanding properties [24,25,26,27,28,29,30], which makes this material very interesting and useful for a wide range of applications [31,32,33,34,35,36]. In contrast to graphene and BNNSs, a TMD monolayer itself contains three layers of atoms (X-M-X), where the transition metal M, for example, molybdenum, is “sandwiched” between two layers of the nonmetal atom X, for example, sulfur in the case of MoS_2_ [20]. Similar to other 2D nanomaterials, TMDs can also be used in a wide variety of applications [37,38,39,40,41,42] due to their properties [38,43,44,45,46,47]. In addition, 2D nanosheets have the potential to revolutionise technology. However, the large-scale production of these materials still remains a major challenge. A very promising method is the liquid-phase exfoliation (LPE) because it allows the possibility of upscaling and increasing the yields [22,48,49,50,51]. Many articles on understanding the mechanism of LPE and advancing the yields of 2D materials have already been published [52,53,54,55,56,57,58,59], The sonication-assisted LPE is a method in which layered materials are exfoliated by concentrated energy that is released in the dispersion due to the cavitational implosion of the ultrasound [60]. In the right solvent, by minimising the interfacial tension, the van der Waals force between the layers can be overcome, and the exfoliated nanosheets are dispersed stably in the solvent [51,61]. Liquid cascade centrifugation (LCC) [62,63] has also been shown to be effective in the production of 2D materials with controlled size and thickness but at the cost of low yield. LPE is a promising and powerful method for large-scale production of 2D materials, the obtained yields (0.04% [64] and 3% [48] for graphene, 2% [22] and 2.6% [65] for BNNSs, and 4.8% for MoS_2_ NS [66]) are still low and so far, there has been no explanation as to why. Yuan et al. have reported a yield of 26% for BNNSs by using sonication [67]; however, they start from a hydroxyl-functionalised boron nitride. Hernandez et al. [49] have also reported an increased yield by bath sonication recycling (12% for graphene); however, they use NMP as a solvent. The aim of our study is to increase the yield by using green solvents [68], such as acetone for graphene and 2-propanol for BNNSs and MoS_2_ NS, as well as by starting from their non-modified bulk counterpart, by first understanding the physical–chemical phenomenon that appears in the dispersion during the sonication process. The behaviour of the nanosheets in the solvent provides an insight into the cause of the current low yields. Experimental analyses were performed to investigate and study the production of graphene, BNNSs, and MoS_2_ by using the sonication-assisted LPE method.

## 2. Materials and Methods

The bulk materials used for the production of 2D nanosheets are as follows: graphite was purchased from Alfa Aesar GmbH & Co KG (Karlsruhe, Germany); hexagonal boron nitride (h-BN), purchased from ESK Ceramics (3M Deutschland GmbH, Neuss, Germany); molybdenum disulfide (MoS_2_), purchased from Sigma-Aldrich Chemie GmbH (Steinheim, Germany). The 3D bulk materials are in the μm range. The exfoliation solvents were 2-propanol (purity (GC) ≥ 99.9%) was purchased from Carl Roth GmbH + Co. KG (Karlsruhe, Germany); acetone (purity (GC) ≥ 99.8%), purchased from Carl Roth GmbH + Co. KG (Karlsruhe, Germany); N-methyl-2-pyrrolidone (purity (GC) ≥ 99.5%), purchased from Merck KGaA (Darmstadt, Germany). All solvents were used without further purification. Poly(ethylene oxide) with an M.W of 100,000 was purchased from Sigma-Aldrich Chemie GmbH (Steinheim, Germany).

### 2.1. Probe-Type Sonication

For this procedure, 1 g of the starting bulk material was dispersed in 100 mL 2-propanol and sonicated for a total of 21 h for boron nitride and 26 h molybdenum disulfide, and 1 g of the starting bulk material graphite was dispersed in 100 mL acetone and sonicated for a total of 17 h. The sonication was performed at an amplitude of 30% and with an On/Off pulse of 0.5/0.5 s. The probe-type sonicator that was used was Sonics & Materials, 400 Watt-Model with a variable power of max. 400 W and a frequency of 20 kHz. The probe was a standard horn ½” (13 mm) with a threaded end and replaceable tip ½” (13 mm) and made of titanium alloy (Ti-6Al-4V). The sample was cooled with an ice bath during the sonication process. The experiments were performed in triplicates. Therefore, three small samples were taken every hour, in order to determine the yield and follow the production of the nanosheets, which are separated from their non-exfoliated bulk materials by a 10 min centrifugation at 4500 rpm. The centrifuge that was used was the Heraeus Labofuge 400R with a swinging bucket rotor (max. radius 17.4 cm) from Kendro Laboratory Products GmbH (Osterode, Germany).

### 2.2. Diluting and Stirring

During this stage, 1 g of the starting bulk material was dispersed in 100 mL 2-propanol and sonicated for a total of 21 h for boron nitride and 26 h for molybdenum disulfide, and 1 g of the starting bulk material graphite was dispersed in 100 mL acetone and sonicated for a total of 17 h. The same sonication parameters as above were used. The sample was then diluted with the following dilution ratios: 1:1; 1:10 and 1:100. In the case of graphene, a solvent exchange from acetone to N-methyl-2-pyrrolidone (NMP) was performed. The diluted samples were stirred for one hour and the exfoliated nanosheets were separated by a 10 min centrifugation at 4500 rpm. For comparison, all three materials were also stirred without previous sonication. Parameters such as initial concentration (10 mg/mL) and solvent (2-propanol for boron nitride and molybdenum disulfide and NMP for graphite) were the same as above.

### 2.3. Recycling

Briefly, 100 mg bulk material (boron nitride and molybdenum disulfide) was dispersed in 10 mL 2-propanol, and 100 mg graphite was dispersed in 10 mL acetone. The materials were sonicated for one hour using the same sonication parameters as above, and the exfoliated nanosheets were separated from the bulk material by a 10 min centrifugation at 4500 rpm. The non-exfoliated material was redispersed in fresh solvent (2-propanol for boron nitride and molybdenum disulfide and acetone for graphite) and further sonicated for another hour. This process was repeated for a total of 12 h. 

### 2.4. Enhanced Liquid-Phase Exfoliation

During one cycle, 100 mg bulk material (boron nitride and molybdenum disulfide) was dispersed in 10 mL 2-propanol, and 100 mg graphite was dispersed in 10 mL acetone. The materials were sonicated for one hour using the same sonication parameters as above. They were then diluted with a 1:10 ratio and stirred for one hour. The exfoliated nanosheets were separated from the bulk material by a 10 min centrifugation at 4500 rpm. The non-exfoliated material was redispersed in fresh solvent (2-propanol for boron nitride and molybdenum disulfide and acetone for graphite) and recycled. This process was repeated for a total of 5 cycles. 

### 2.5. Ultraviolet–Visible Spectroscopy (UV–Vis) Measurements

The yield was determined using UV–Vis spectroscopy and the Lambert–Beer law. The photometer that was used was the Aligent Cary 60 Spectrophotometer (Aligent Technologies Österreich GmbH, Vienna, Austria) with the following electrical specifications: standard 3.2 A/12 V plug pack as the main supply; spectrophotometer: 90–265 V AC and a frequency of 47–63 Hz. The absorption coefficients are 2474 L g^−1^m^−1^ at λ = 660 nm for graphene, 2354 L g^−1^m^−1^ at λ = 300 nm for h-BN and 3302 L g^−1^m^−1^ at λ = 672 nm for MoS_2_ [51].

### 2.6. Dynamic Light Scattering (DLS) Measurements

The instrument comprised a goniometer, a diode laser working at λ = 532 nm (Coherent Verdi V5) with single fiber detection optics (OZ from GMP, Zürich, Switzerland), an ALV/SO-SIPD/DUAL photomultiplier with pseudo-cross-correlation mode, and an ALV 7004 digital multi-tau real-time correlator (ALV, Langen, Germany). The AVL software package was used to record and store the correlation functions. These were averaged with 10 measurements of 30 s at a scattering angle of 90° and a temperature of 25 °C. The hydrodynamic radius was calculated by the optimised regulation technique software [69].

### 2.7. Small Angle X-ray Scattering (SAXS) Measurements

The SAXS instrument consisted of a SAXSpoint 2.0 (Anton-Paar GmbH, Graz, Austria) that contained a Primux 100 micro microfocus X-ray source operating at λ = 0.154 nm (Cu Kα). The samples were filled into a 1 mm diameter capillary and measured 10 times, for 180 s. Two-dimensional scattering patterns that were recorded by a 2D EIGER series hybrid photon counting (HPC) detector (Dectris Ltd., Baden-Daettwil, Switzerland), were averaged and edited by correcting the cosmic X-ray impacts. All measurements were performed at 20 °C. Water was used as a secondary standard in order to achieve the absolute scale calibration [70]. All SAXS data have been evaluated by a generalised indirect Fourier-transform (GIFT) method [71,72,73] to determine the pair distance distribution function of the thickness pt(r) [74].

### 2.8. Atomic Force Microscopy (AFM) Measurements

The instrument used was the atomic force microscope Tosca^TM^ 400 (Anton-Paar GmbH, Graz, Austria) with a power supply of 100 to 240 V ± 10%, frequency of 50 to 60 Hz, power consumption of 200 W, and fuse T 3.6 AH. The tapping mode was used for imaging the sample surface at 10 μm resolution. The cantilever had a force constant of 42 N and a resonance frequency of 285 kHz.

## 3. Results

Despite the liquid-phase exfoliation being a promising method for the large-scale production of 2D materials, yields achieved by continuous sonication in previous studies are low. Understanding what occurs during the sonication process would be the first step towards increasing the yields.

### 3.1. Probe-Type Sonication

**Figure 1:**
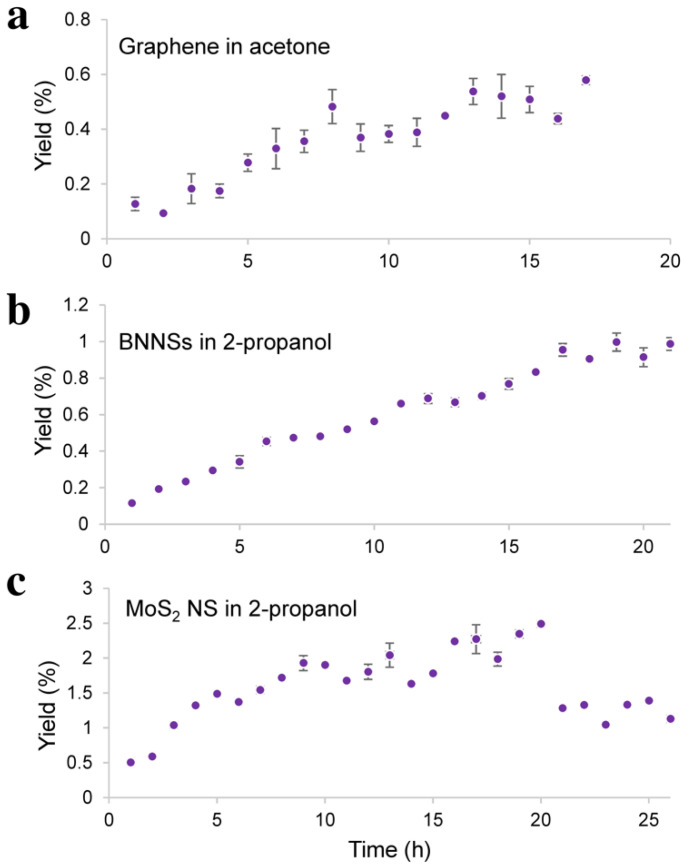
(**a**) Production of graphene in acetone, (**b**) BNNSs in 2-propanol and (**c**) MoS_2_ NS in 2-propanol by sonication-assisted liquid-phase exfoliation. The maximum yield obtained for graphene was 0.5%, and it was reached after 12 h of sonication. The BNNSs reached a maximum yield of 0.9% after 17 h of sonication, and the maximum yield for MoS_2_ NS was 2.3% after 16 h of sonication.

Graphene, BNNSs, and MoS_2_ NS were exfoliated by probe-type sonication from their bulk counterparts graphite, h-BN, and MoS_2_. The obtained yields are shown in Figure 1.

The yield of the exfoliated 2D materials was initially increased with sonication time. However, longer sonication times led to a flattening of the curve. The maximum yield obtained for graphene was 0.5%, and it was reached after 12 h of sonication, as shown in Figure 1a. The BNNSs reached a yield of 0.9% after 17 h of sonication, as shown in Figure 1b, and the maximum yield of MoS_2_ NS was 2.3%, and it was reached after 16 h of sonication, as shown in Figure 1c. The sonication time needed to achieve maximum yields varied depending on the material and the exfoliation solvent. The different molecular structures led to different amounts of energy that were necessary in order to exfoliate the nanosheets from their bulk material. The chosen solvent also had an effect on the exfoliation and stabilisation of the nanosheets. 2-Propanol is a promising solvent [22] and has shown a high exfoliation efficiency for boron nitride and molybdenum disulfide. In the case of graphene, N-methyl-2-pyrrolidone (NMP) would have been a good solvent, but it could not be used for the probe-type sonication because of its sonochemical degradation [75]. Therefore, acetone was chosen as an exfoliation solvent, because the ratio of surface tension components (polar component/dispersive component) of acetone is closest to that of NMP as demonstrated by Shen et al. [51]. Regardless of different sonication times needed to achieve the maximum yield, one common behaviour observed for all three materials was that there was no significant increase in the yield after a certain concentration of the nanosheets in the sample was reached. Furthermore, in the case of MoS_2_ NS, the yield was decreased. This behaviour was also found in other studies [76,77,78,79]. Thus far, no explanation why this occurs has been reported. This raises the question of whether the exfoliation of nanosheets from their bulk counterpart stops despite further sonication, or whether they are continuously being exfoliated from the bulk material but reaggregate.

### 3.2. Diluting and Stirring

**Figure 2:**
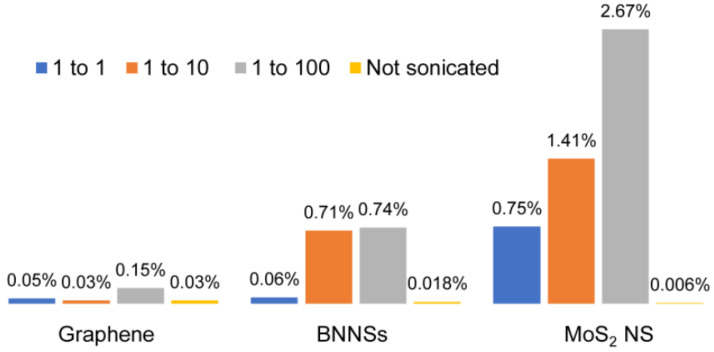
Production of graphene in NMP, BNNSs in 2-propanol, and MoS_2_ NS in 2-propanol by reducing the concentration of the exfoliated nanosheets in dispersion and stirring for one hour. The maximum yields achieved were 0.15% for graphene, 0.74% for BNNSs, and 2.67% for MoS_2_ NS. These results were higher, compared with those without a previous sonication.

To determine if the reaggregation is reversible, after removing the exfoliated nanosheets, the samples were diluted and stirred after the sonication process, and the yield was determined via UV–Vis spectroscopy. Three different dilution ratios were used, and they were as follows: 1:1; 1:10, and 1:100. The samples were stirred for one hour, and the yields were determined and compared with the ones when the material was stirred without a pre-sonication process. The results are demonstrated in Figure 2.

Stirring the sample without a pre-sonication process showed negligible exfoliation of nanosheets, as shown in Figure 2, where the yields for graphene, BNNSs, and MoS_2_ NS were 0.03%, 0.018%, and 0.006%, respectively. However, stirring the sample after it was sonicated for a few hours led to an increase in yield. In the case of graphene, this was very low, almost non-significant, with values such as 0.05%, 0.03%, and 0.15%. The reason for this might be the overall lower efficiency of acetone in comparison with NMP as an exfoliation solvent for this material, as the maximum yield of graphene obtained after 12 h of sonication was only 0.5%. Although for the stirring process, a solvent exchange from acetone to NMP was performed, this did not seem to have an effect on yield. The results were more promising for boron nitride nanosheets and molybdenum disulfide nanosheets, with yields up to 0.74% and 2.67%, respectively.

### 3.3. Recycling

**Figure 3:**
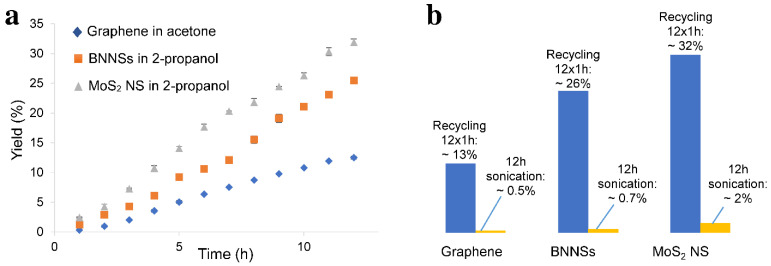
(**a**) Production of graphene in acetone, BNNSs in 2-propanol, and MoS_2_ NS in 2-propanol by recycling. The maximum yields achieved after a total of 12 h of sonication were 12.5% for graphene, 25.5% for BNNSs, and 31.9% for MoS_2_ NS; (**b**) comparison of the yields achieved by recycling to those achieved by 12 h of sonication. A total yield of 12.5% was achieved for graphene by recycling, whereas without recycling the yield was 0.45% after 12 h of sonication. In the case of BNNSs, the values were 25.5% for recycling and 0.69% after 12 h of sonication, and in the case of MoS_2_ NS, the yield was as high as 31.9% after recycling and only 1.8% when the sample was sonicated for 12 h straight.

Recycling the bulk material led to an increase in yield, as illustrated in Figure 3.

The exfoliated nanosheets were removed from the sample after one hour of sonication, and the bulk material was redispersed in the same solvent (acetone for graphite and 2-propanol for the boron nitride and molybdenum disulfide) and sonicated for another hour. This process was repeated for a total of 12 h. A linear increase in yield was observed for all three materials. In the case of graphene, a total yield of 12.5% was achieved after 12 h of recycled sonication, whereas for BNNSs, the yield was 25.5%, and for MoS_2_ NS, 31.9%. These results are significantly higher, compared with those found in literature (0.04% [64] and 3% [48] for graphene; 2% [22] and 2.6% [65] for BNNSs; 4.8% [66] for MoS_2_ NS), obtained by single-step procedures. Due to its linear character, the yield can be further increased by continuous recycling.

### 3.4. Induced Flocculation

**Figure 4:**
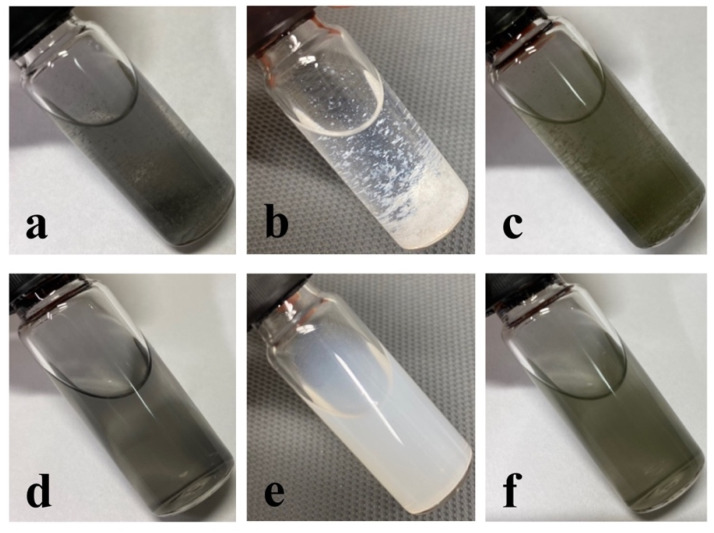
Induced flocculation of (**a**) graphene, (**b**) BNNSs and (**c**) MoS_2_ NS by adding poly(ethylene oxide); (**d**–**f**) this flocculation can be reversed by shaking the sample.

To demonstrate that the flocculation is reversible, the depletion interaction concept was investigated [80,81,82]. Adding a polymer to the dispersion provoked an attraction between the particles, leading to induced flocculation [83]. Poly(ethylene oxide) was added to a dispersion of Graphene, BNNSs, and MoS_2_ NS, and after a certain amount of time, flakes were observed in solution, as shown in Figure 4. The flocculated nanosheets were then successfully redispersed in a solvent by shaking the sample.

### 3.5. Enhanced Liquid-Phase Exfoliation

**Figure 5:**
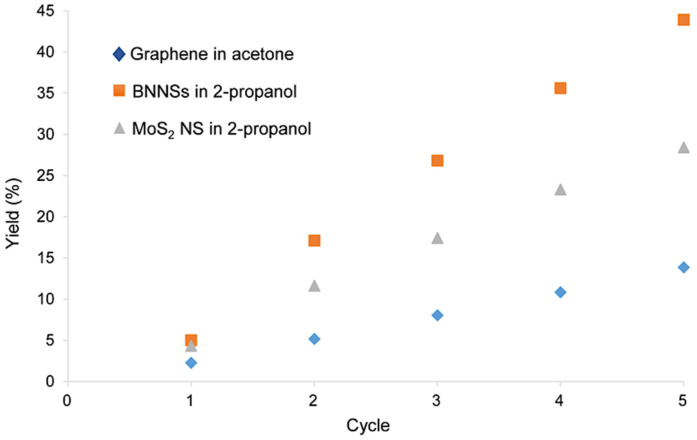
Total yields after five cycles of proposed enhanced LPE: 14% for graphene, 44% for BNNSs, and 29% for MoS_2_ NS.

By combining diluting and stirring with recycling, we were able to enhance the liquid-phase exfoliation method and increase the yield to 14% for graphene, 44% for BNNSs, and 29% for MoS_2_ NS after only five cycles, as illustrated in Figure 5.

During one cycle, the starting bulk material was sonicated for one hour, and after the sample was diluted and stirred for another hour, the exfoliated nanosheets were removed and the bulk material recycled. Although according to Figure 2, a dilution ratio of 1:100 would be a better condition, here, a ratio of 1:10 was chosen in order to reduce the amount of solvent used, as more than one cycle was performed. After five cycles, a remarkable increase in yield was observed (Figure 5). Its linear character, determined by the linear relationship formulas y = 2.88x − 0.63 (R^2^ = 0.9999) for graphene; y = 9.63x − 3.21 (R^2^ = 0.9941) for BNNSs; y = 5.99x − 0.97 (R^2^ = 0.996) for MoS_2_ NS, indicates the continuation of the production of 2D materials can lead to even higher yields.

### 3.6. Characterisation

**Figure 6:**
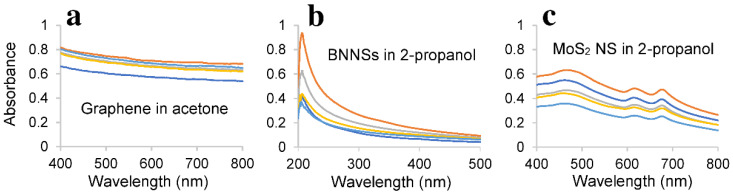
UV–Vis spectra of (**a**) graphene, (**b**) BNNSs, and (**c**) MoS_2_ NS produced by five cycles of enhanced LPE.

The yield was determined using UV–Vis spectroscopy and the Lambert–Beer law (A = ε l c, where A is the absorbance, ε is the absorption coefficient, l is the optical path length in cm and c is the concentration). The UV–Vis spectra of graphene, BNNSs, and MoS_2_ NS produced by five cycles of enhanced LPE are illustrated in Figure 6. 

The concentration of the nanosheets in the sample was calculated by the absorbance value at the specific wavelengths (λ = 660 nm for graphene, λ = 300 nm for BNNSs and λ = 672 nm for MoS_2_ NS), divided by the corresponding absorption coefficients, which are 2474 L g^−1^m^−1^ at λ = 660 nm for graphene; L g^−1^m^−1^ at λ = 300 nm for h-BN; 3302 L g^−1^m^−1^ at λ = 672 nm for MoS_2_ [51].

The average hydrodynamic radius of the exfoliated nanosheets was characterised by dynamic light scattering (DLS), whereas their maximum thickness was determined by small-angle X-ray scattering (SAXS). DLS and SAXS could only be performed on those exfoliated nanosheets that were produced by long probe-type sonication and recycling. Due to the low yield of those produced by stirring after sonication, the scattering intensity of the nanosheets was too weak, and therefore, it could not be measured. Moreover, SAXS was used to determine the maximum thickness of the nanosheets. The low scattering intensity of thin flakes and monolayers was superposed by the much higher scattering intensity of the thicker ones.

**Figure 7:**
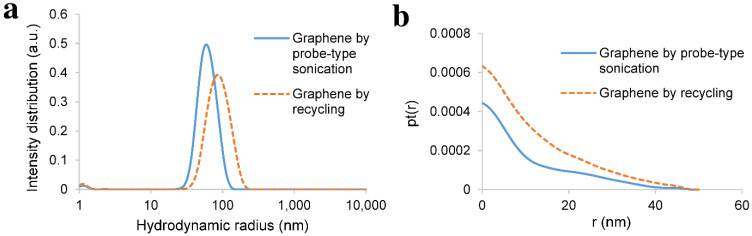
(**a**) DLS: hydrodynamic radius of the exfoliated graphene by probe-type sonication and by recycling. Graphene that was exfoliated by long probe-type sonication has an average hydrodynamic radius of around 65 nm, whereas graphene that was sonicated for one hour in the case of recycling has an average hydrodynamic radius of around 100 nm; (**b**) SAXS: pair–distance distribution function of thickness (pt(r), where r (nm) stands for thickness of the nanosheets) of the exfoliated graphene by probe-type sonication and by recycling. Graphite nanosheets exfoliated by long probe-type sonication had a maximum thickness of around 15 nm and could increase to 40 nm, whereas graphite nanosheets that were sonicated for one hour in the case of recycling had a maximum thickness of 20 nm and could increase to 40 nm.

**Figure 8:**
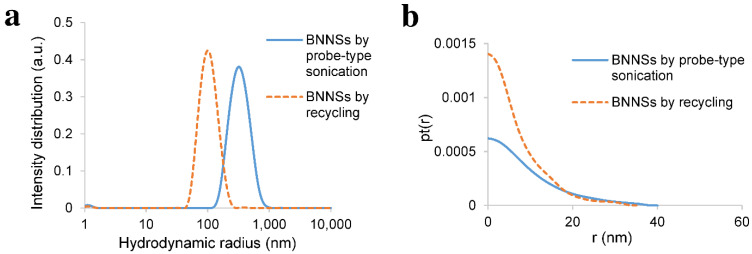
(**a**) DLS: hydrodynamic radius of the exfoliated BNNSs by probe-type sonication and by recycling. BNNSs exfoliated by long probe-type sonication had an average hydrodynamic radius of around 350 nm, whereas BNNSs sonicated for one hour in the case of recycling had an average hydrodynamic radius of around 110 nm; (**b**) SAXS: pair–distance distribution function of thickness (pt(r), where r (nm) stands for thickness of the nanosheets) of the exfoliated BNNSs by probe-type sonication and by recycling. BNNSs exfoliated by long probe-type sonication had a maximum thickness of around 20 nm and could increase to 30 nm, whereas BNNSs sonicated for one hour in the case of recycling had a maximum thickness of 15 nm and could rise to 30 nm.

As illustrated in Figure 7a, the average hydrodynamic radius of graphene exfoliated by probe-type sonication was around 65 nm, whereas the graphene that was exfoliated by recycling, where graphite was sonicated for only one hour, showed a hydrodynamic radius of around 100 nm. Monolayer and few-layer graphene could not be determined by SAXS due to their low scattering intensity. However, the maximum thickness of the graphite nanosheets exfoliated by continuous probe-type sonication was between 15 nm and 40 nm. Similarly, graphite nanosheets that were exfoliated by recycling showed maximum thickness between 20 nm and 40 nm (Figure 7b). A more significant difference in hydrodynamic radius was observed in the case of BNNSs, as illustrated in Figure 8a.

The average hydrodynamic radius of BNNSs exfoliated by only one hour of sonication in the case of recycling was approximately 110 nm, whereas the long sonication process led to a larger hydrodynamic radius, which was around 350 nm. However, comparable to graphene, the maximum thickness does not change significantly. The BNNSs that were exfoliated after one hour of sonication in the case of recycling showed a maximum thickness of up to 15–30 nm, whereas those exfoliated by long probe-type sonication had a maximum thickness of up to 20–30 nm (Figure 8b).

**Figure 9:**
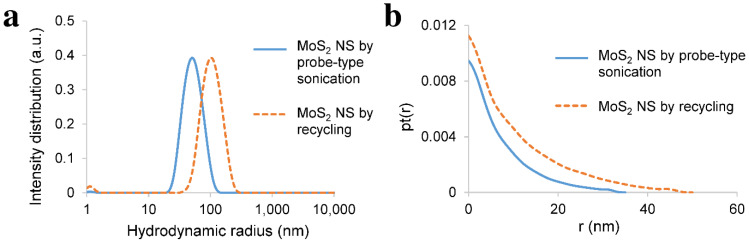
(**a**) DLS: hydrodynamic radius of the exfoliated MoS_2_ NS by probe-type sonication and by recycling. MoS_2_ NS that was exfoliated by long probe-type sonication had an average hydrodynamic radius of around 60 nm, whereas MoS_2_ NS that was sonicated for one hour in the case of recycling had an average hydrodynamic radius of around 110 nm; (**b**) SAXS: pair–distance distribution function of thickness (pt(r), where r (nm) stands for thickness of the nanosheets) of the exfoliated MoS_2_ NS by probe-type sonication and by recycling. MoS_2_ NS exfoliated by long probe-type sonication had a maximum thickness of around 10 nm and could increase to 30 nm, whereas MoS_2_ NS that was sonicated for one hour in the case of recycling had a maximum thickness of 15 nm and could increase to 40 nm.

Similar to graphene, MoS_2_ NSs that were produced by long probe-type sonication had an average hydrodynamic radius of around 60 nm, whereas those produced by one hour of sonication in the case of recycling had an average hydrodynamic radius of about 110 nm, as demonstrated in Figure 9a. However, SAXS showed that the nanosheets produced by long sonication were thinner than those exfoliated by one hour of sonication (Figure 9b). The nanosheets produced by long probe-type sonication had maximum thicknesses of up to 10 nm to 30 nm, while those that were produced by one hour of sonication in the case of recycling had maximum thicknesses of up to 15–40 nm. In the case of MoS_2_ NS, longer sonication led to a smaller hydrodynamic radius and thinner nanosheets. Nevertheless, the effect of the sonication time on the size of the exfoliated 2D materials needs further investigation.

**Figure 10:**
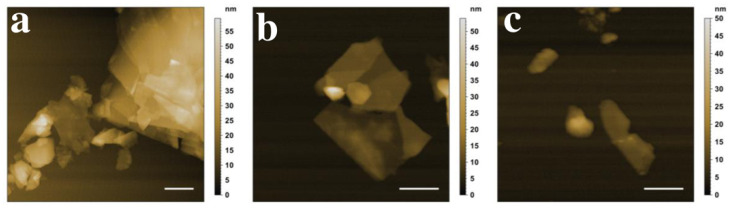
AFM images of the exfoliated (**a**) graphene, (**b**) BNNSs, and (**c**) MoS_2_ NS. Scale bar (white): 250 nm.

The quality of the nanosheets was determined via AFM, as demonstrated in Figure 10.

The AFM images illustrated a typical 2D shape of the exfoliated nanosheets with different sizes and thicknesses, as it is challenging to produce monodisperse samples by LPE. Monolayer and few-layer graphene with a thickness of under 6 nm, as well as graphite nanosheets with a thickness of up to 25 nm, are visible in Figure 10a. The BNNSs in Figure 10b also showed different thicknesses, ranging from 4 nm to 15 nm, whereas Figure 10c illustrates MoS_2_ NS that were similar in their thickness, around 8–9 nm.

## 4. Discussion

**Figure 11:**
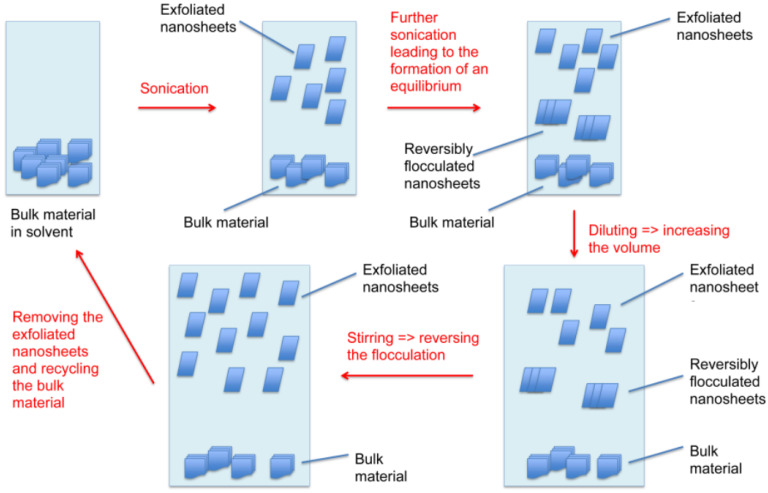
Improved LPE method in order to increase the yield. When the equilibrium in the sample was reached, and flocculated nanosheets were created, the sample was diluted and stirred in order to redisperse the flocculated nanosheets. The exfoliated 2D material was then removed, the bulk material was recycled, and the process was repeated.

To overcome the challenge of producing 2D materials at a large scale, it is of high significance to first understand what occurs during the sonication-assisted liquid-phase exfoliation, and why the overall yields are low. As shown in Figure 1, the yields increased with sonication time; however, when a certain concentration of the nanosheets in the sample was achieved, the curve flattened, reaching a plateau. We speculate that during sonication, exfoliation and reaggregation were in equilibrium. With the increase in the concentration of nanosheets, the distance between them became smaller. The closer they were, the stronger the attractive van der Waals forces, and this led to their flocculation. The cohesion in those aggregates was weaker than in the pristine materials. Although a little-to-no increase in yield was observed after several hours of sonication, as shown in Figure 1, the exfoliation of nanosheets from their bulk material continued. While these nanosheets were being produced, those that were already exfoliated flocculated back together due to the decrease in the distance between them. This led to a concentration of 2D materials in the sample that stayed almost constant despite continuous sonication. After several hours, equilibrium was reached. This equilibrium between the exfoliated nanosheets and the flocculated ones, prevented the further increase in yield, as demonstrated in Figure 1 by the flattening of the curve. Nevertheless, due to weaker cohesion in the flocculated nanosheets, they could be redispersed by diluting and stirring the sample. The reversibility was demonstrated by the induced flocculation shown in Section 3.4. Furthermore, the presence of nanosheets when the sample was stirred after the sonication process (Figure 2) also showed that this flocculation could be reversed. Understanding the equilibrium state that occurred during the sonication-assisted LPE provided us an insight into the cause of low yields, despite long hours of sonication. Another fact that supports the formation of this apparent equilibrium is that the yield could be increased by recycling. Continuous sonication did not increase yield; however, when the exfoliated nanosheets were removed, and the bulk material was recycled, the equilibrium could be avoided, and more nanosheets could be exfoliated. A comparison of the yields achieved by recycling with those achieved by a long 12 h sonication is illustrated in Figure 3b. The yields that were obtained by recycling procedure and those obtained by continuous sonication were approximately 28 times higher in the case of graphene, 37 times higher in the case of BNNSs, and 18 times higher in the case of MoS_2_ NS. This significant increase in yield supports the formation of an apparent equilibrium during sonication that prevented the continual production of nanosheets. Therefore, it was necessary to remove the already exfoliated nanosheets and recycle the bulk material to further exfoliate and consequently increase the total yield. On the basis of these results, we developed a method by combining the recycling method with diluting and stirring, as illustrated in Figure 11.

By diluting and stirring the sample after sonication, the flocculated nanosheets were redispersed. This led to a higher amount of exfoliated nanomaterial in the sample. The nanosheets were then removed by centrifugation, and the bulk material was recycled. This multi-step procedure significantly increased the yield. Following this scheme, we were able to achieve yields of graphene, BNNSs, and MoS_2_ NS to 14%, 44%, and 29%, respectively, after only five cycles (Figure 5). This five-cycle LPE mechanism, shown in Figure 11, supports the fact that the formation of the apparent equilibrium during sonication prevents the further production of nanosheets. It can be circumvented by diluting and stirring, as well as recycling the non-exfoliated material. In a subsequent step, the nanosheets can be separated by high-speed centrifugation, and the solvent can be reused in this procedure. Due to the fact that yields can be increased by avoiding the equilibrium, this method can be extended to the production of different 2D materials, as well as the use of other solvents. The results in Figure 5 demonstrate that the proposed procedure leads to the continual production of nanosheets. Therefore, the LPE method demonstrated in Figure 11 can be repeated continuously leading to even higher yields.

## 5. Conclusions

Understanding what occurs during the sonication-assisted LPE is an important step towards the large-scale production of 2D materials. Although this method is very promising, the obtained yields that were previously reported were still low. We were able to investigate and show the reasons behind these low yields. In this report, we showed that during sonication, exfoliated nanosheets and the flocculated ones are in equilibrium. When a certain concentration of 2D materials in the sample is achieved, the distance between them becomes smaller, leading to their flocculation. However, the experiments showed that this is reversible. By combining diluting and stirring (to reverse the flocculation) with recycling (to increase the yield), we developed an LPE procedure (Figure 11) circumventing the equilibrium in order to increase yield. After only five cycles, we were able to achieve yields of 14% for graphene, 44% for BNNSs, and 29% for MoS_2_ NS. Due to similarities in structure, our findings can be extended to other 2D materials as well.

## Data Availability

The data is included in the main text.
